# Analysis of apyrase 5' upstream region validates improved *Anopheles gambiae *transformation technique

**DOI:** 10.1186/1756-0500-2-24

**Published:** 2009-02-19

**Authors:** Fabrizio Lombardo, Gareth J Lycett, Alessandra Lanfrancotti, Mario Coluzzi, Bruno Arcà

**Affiliations:** 1 Department of Public Health – Parasitology Section, "Sapienza" University of Rome, Piazzale Aldo Moro 5, 00185, Roma, Italy; 2 European Molecular Biology Laboratory, Meyerhofstrasse 1, 69117-Heidelberg, Germany; 3 Department of Structural and Functional Biology, University Federico II, Via Cinthia, 80126, Napoli, Italy; 4 Imperial College London, Imperial College Road, SW7 2AZ, London, UK; 5 Liverpool School of Tropical Medicine, Pembroke Place, L3 5QA, Liverpool, UK; 6 Kennedy Institute of Rheumatology, Imperial College London, 1 Aspenlea Road, W6 8LH, London, UK

## Abstract

**Background:**

Genetic transformation of the malaria mosquito *Anopheles gambiae *has been successfully achieved in recent years, and represents a potentially powerful tool for researchers. Tissue-, stage- and sex-specific promoters are essential requirements to support the development of new applications for the transformation technique and potential malaria control strategies. During the *Plasmodium *lifecycle in the invertebrate host, four major mosquito cell types are involved in interactions with the parasite: hemocytes and fat body cells, which provide humoral and cellular components of the innate immune response, midgut and salivary glands representing the epithelial barriers traversed by the parasite during its lifecycle in the mosquito.

**Findings:**

We have analyzed the upstream regulatory sequence of the *An. gambiae *salivary gland-specific *apyrase *(*AgApy*) gene in transgenic *An. gambiae *using a *piggyBac *transposable element vector marked by a *3xP3 *promoter:*DsRed *gene fusion. Efficient germ-line transformation in *An. gambiae *mosquitoes was obtained and several integration events in at least three different G_0 _families were detected. *LacZ *reporter gene expression was analyzed in three transgenic lines/groups, and in only one group was tissue-specific expression restricted to salivary glands.

**Conclusion:**

Our data describe an efficient genetic transformation of *An. gambiae *embryos. However, expression from the selected region of the *AgApy *promoter is weak and position effects may mask tissue- and stage- specific activity in transgenic mosquitoes.

## Background

### 

The mosquito *Anopheles gambiae *is the main vector of the human malaria parasite *Plasmodium falciparum *in sub-Saharan Africa. Within the insect, the parasite undergoes a complex life-cycle that includes fertilization, midgut invasion, sporozoite maturation, avoidance of the mosquito innate immune response and, as prerequisite for a successful transmission, recognition and entrance into the salivary glands [1]. The development of tools for mosquito genetic manipulation have provided evidence that *Plasmodium *development can be modified in the anopheline vector and opened new perspectives for studies on vector biology and on parasite-vector-host interactions [2,3].

Several studies in the last decade reported the successful use of tissue-specific promoters for directing the expression of exogenous genes in different mosquito target organs (primarily midgut, hemocoel and salivary glands), mainly in the yellow fever vector *Aedes aegypti *and in the Asian malaria vector *Anopheles stephensi *[4-7]. As far as the main African malaria vector *An. gambiae *is concerned, after the initial successful transformation [8] only one additional study with transgenic *An. gambiae *has been reported so far [9]. In both cases, low transformation efficiencies were observed.

One of our specific interests has been the analysis of *An. gambiae *salivary gland-specific promoters. We have previously analyzed the putative promoter regions of the *An. gambiae *salivary gland-specific *D7-related 4 *(*D7r4*) and *apyrase *(*AgApy*) genes in the fruitfly and in *An. stephensi *[10-12]. We reported that a short region (~800 bp) from the *An. gambiae AgApy *promoter was able to drive stage- and tissue-specific expression of the reporter gene in transgenic *An. stephensi*. Compared to the endogenous expression pattern of the *AgApy *gene in *An. gambiae*, however, the level of expression in transgenic *An. stephensi *was low and the transgene was expressed in the proximal-lateral rather than distal-lateral lobes [12,13]. We concluded that additional regulatory information, possibly located upstream, was missing in the short fragment used. It was also clear that the putative *An. gambiae *promoter needed to be examined directly in this species before more firm conclusions could be drawn. In this context, we should mention that while this work was in progress, robust salivary gland specific expression of a reporter gene in *An. stephensi *was reported using a promoter fragment from the *An. stephensi aapp *gene [14].

## Methods

If not otherwise indicated, experimental procedures were according to Sambrook and colleagues [15]. The sequences of the oligonucleotide primers used in this study are listed in the Additional file 1.

### Plasmid construction

pBac(3xP3RED)AgApy was constructed by amplification of a 2454 bp fragment from the *AgApy *promoter followed by cloning into a shuttle bluescript-based vector upstream of the *E. coli LacZ *coding region and the bovine growth hormone (*bgh*) terminator. The resulting cassette was transferred into the pSLfa1180fa plasmid vector [16] and then inserted in the pBac [3xP3-DsRed] vector containing the *DsRed *coding sequence regulated by 3xP3 promoter and SV40 terminator [17]. The resulting pBac(3xP3RED)AgApy construct was purified using the QIAGEN Plasmid Midi kit (QIAGEN, Germany), sequenced and used for embryo microinjection after mixing with phspBac [18], ethanol precipitation and resuspension at 500 μg/ml (3.5:1.5 vector:transposase).

### *An. gambiae *germline transformation

Embryonic injections were performed essentially as described by Lobo N.F. and colleagues [19]. The detailed protocol used to perform *An. gambiae *embryos injections is reported in the Additional file 2.

### Southern hybridization

Eight micrograms of genomic DNA from each transgenic group were digested with *Hind*III, fractionated on a 0.8% agarose gel and transferred on to a nylon membrane. Hybridization and washings were performed at 65°C under high stringency conditions. The pBac probes (pBacL and pBacR, spanning the pBac arms) were obtained by PCR, whilst the *AgApy *1,8 kbp promoter fragment used as probe was obtained by *EcoR*I digestion of the pBac(3xP3RED)AgApy transformation plasmid.

### RNA Extraction and RT-PCR

Total RNA was extracted using the TRIZOL Reagent (Invitrogen, Carlsbad, CA, USA), treated with RNase free-DNaseI (Invitrogen) and approximately 80 ng used to synthesize cDNA for PCR amplification using the SuperScript one-step RT-PCR system (Invitrogen) according to manufacturer instructions. Reverse transcription (50°C, 30 minutes) and heat inactivation of the reverse transcriptase (94°C, 2 minutes) were followed by 25 (*rpS7 *mRNA) or 35 (*LacZ *and *DsRed *mRNA) PCR cycles: 30 seconds at 94°C, 30 seconds at 55°C, 1 minute at 72°C. Control PCR amplifications without the reverse transcription step were also performed. All the reactions were performed at least twice using different batches of RNA preparations.

## Results and discussion

**Figure 1:**
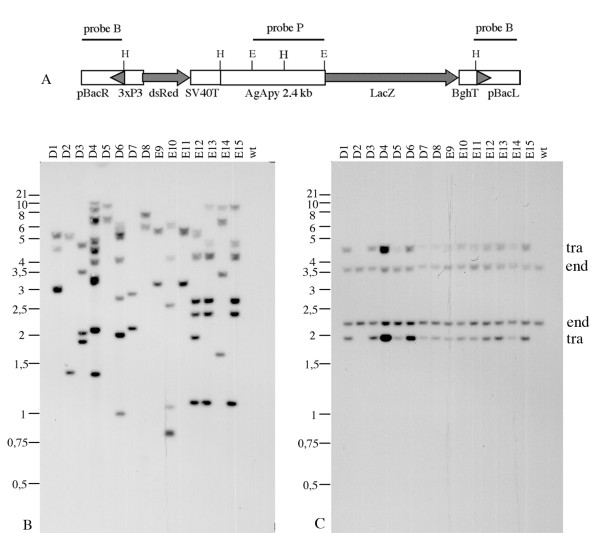
**Transformation construct and Southern blot hybridization**. (A) Schematic representation of the transformation vector pBac(3xP3RED)AgApy used for the *An. gambiae *germ line transformation. The piggyBac left (pBacL) and right (pBacR) arms, the 3xP3 promoter, the *DsRed *transformation marker, the SV40 terminator, the *AgApy *promoter, the *LacZ *reporter gene and the *bgh *terminator are shown. Bars represent the hybridization probes: probe B, hybridizing to the left and right inverted terminal repeats, and probe P, corresponding to a fragment of the *AgApy *promoter. E and H indicate *Eco*RI and *Hind*I restriction sites. (B and C) *Hind*III digested genomic DNA from the different *An. gambiae *transgenic lines are indicated on the top. (B) Hybridization with probe B (Fig. 1A), which detects the piggyBac arms: each insertion is expected to yield two bands of variable size. (C) Hybridization with probe P (Fig. 1A), which detects the *AgApy *promoter: here, each insertion is expected to yield four bands of fixed size irrespective of transgene copy number, two from the endogenous *AgApy *gene (end) and two from the transgene (tra). The numbers on the left refer to the molecular weight marker (Kbp).

We describe here the *in vivo *analysis of a large 5' regulatory region taken from the *AgApy *gene of *An. gambiae*. A fragment of ~2.4 kb, including the short 5'UTR (16 nucleotides) of *AgApy *was ligated in front of the *LacZ *reporter gene and the *bgh *terminator. This expression cassette was inserted into the *3xP3*/*DsRed *marked *piggyBac *vector [17] and the resultant plasmid (Fig. 1A) used to generate transgenic *An. gambiae*.

In three injection periods carried out over consecutive days on egg batches taken from the same pool of blood fed adults, 796 embryos were injected. From these, 102 larvae hatched and 71 G_0 _adults (8.9%, 35 males and 36 females) emerged (Table 1). Selection of transformants was carried out following mating G_0 _adults in small groups. Briefly, according to the day of emergence five male and three female pools (A to H) were established and out-crossed to the parental wild type strain. Screening of the G_1 _progeny for *DsRed *expression in early larval stages allowed the identification of two pools yielding fluorescent progeny: pool D (15 G_0 _females) and pool E (8 G_0 _males) (Table 2). After the first oviposition, the nine surviving females of group D were forced to lay eggs individually, allowing the identification within this pool of at least two independent founders. In total, groups D and E yielded 48 fluorescent larvae out of approximately 1000 screened (~4.8%) and 223 fluorescent larvae out of ~3500 screened (~6.3%), respectively (Table 2). G_1 _progenies were grouped considering the different depositions and the independent founders, when possible (D group), and then were out-crossed to the wt strain. Single ovipositions from fifteen females of the different G_1 _groups were screened in order to identify, if present, different insertions. Indeed, the pattern and the intensity of the red fluorescence in G_2 _individuals showed variability between and within pools D and E, suggesting the presence of different integration events, in terms of both position and/or number (data not shown).

**Table 1:** Injected embryos, hatched larvae and G_0 _adults

Injection	Embryos	Larvae	G_0 _adults
Inj. 1	184	38 (20,6%)	17
Inj. 2	336	18 (5,3%)	14
Inj. 3	276	46 (16,6%)	40

Total	796	102 (12,8%)	71 (8,9%)

**Table 2:** Mating and screening strategy

Pools G_0_*	f/wt G_1_^§^
A (4 M)	0/1800 (3)
B (5 F)	0/800 (3)
C (7 M)	0/3000 (3)
D (15 F)	48/1000 (2)
E (8 M)	223/3500 (3)
F (9 M)	0/4000 (3)
G (16 F)	0/700 (3)
H (7 M)	0/2400 (3)

Total (71: 35M, 36F)	271/16700

* Mating groups (A-H) and number of G_0 _males (M) or females (F) per group are indicated.

^§ ^Number of fluorescent (f) and wild type (wt) G_1 _larvae found during the screening. The number of ovipositions per each mating group is indicated in brackets.

Southern blot hybridization was performed on G_3 _progeny from each of the 15 transgenic groups. This analysis distinguished 13 different genotypes, the majority (8 out of 13) of which corresponded to multiple insertions (Fig. 1B). More specifically, five lines (D2, D5, D7, D8 and E9 = E11) showed a single integration of the transgene; four groups (D1, D3, E10 and E14) carried a double integration; three groups (D6, E12 and E13 = E15) included three copies of the transgene and one (D4) had four or more integrations. Hybridization with a labeled region of the *AgApy *promoter (probe P, Fig. 1A) indicated the presence of fragments of the expected size both for the endogenous and recombinant *AgApy *promoter (Fig. 1C) in virtually all cases. The only exception was line D2 in which the recombinant promoter was not detected, suggesting that transgene rearrangement, involving loss of this region, most likely took place. In the remaining 14 genotypes, the expected correlation between transgene copy number, as estimated from the total of transposon arms (Fig. 1B), and intensity of signal corresponding to the recombinant *AgApy *promoter (Fig. 1C), was observed.

Three transgenic groups carrying alternative numbers and sites of transgene insertion were selected for further analysis: the E9 line, with a single insertion, and groups D4 and D6, carrying multiple copies of the transgene. It should be noted that the selection of groups was influenced significantly by the loss, shortly after initial selection, of a number of the thirteen genotypes originally obtained.

Analysis of the three transgenic lines revealed that beta-galactosidase activity was not detectable using colorimetric assays in either salivary glands or carcasses of adult females. In addition, immuno-staining of whole female salivary glands and western blot analysis of salivary gland extracts both failed to detect beta-galactosidase protein (data not shown).

*LacZ *reporter gene expression analysis was therefore performed by RT-PCR. Initially, the primers LacZF1 and LacZR2, previously employed to characterize *LacZ *expression in transgenic *An. stephensi*, were used [12]. However, amplifications indicated significantly lower expression levels in transgenic *An. gambiae *(see Additional file 3), explaining also the inability to detect beta-galactosidase activity or protein in these lines. For this reason, a novel, better performing primers pair (LBF and LBR) was selected and employed for the following RT-PCR amplifications.

**Figure 2:**
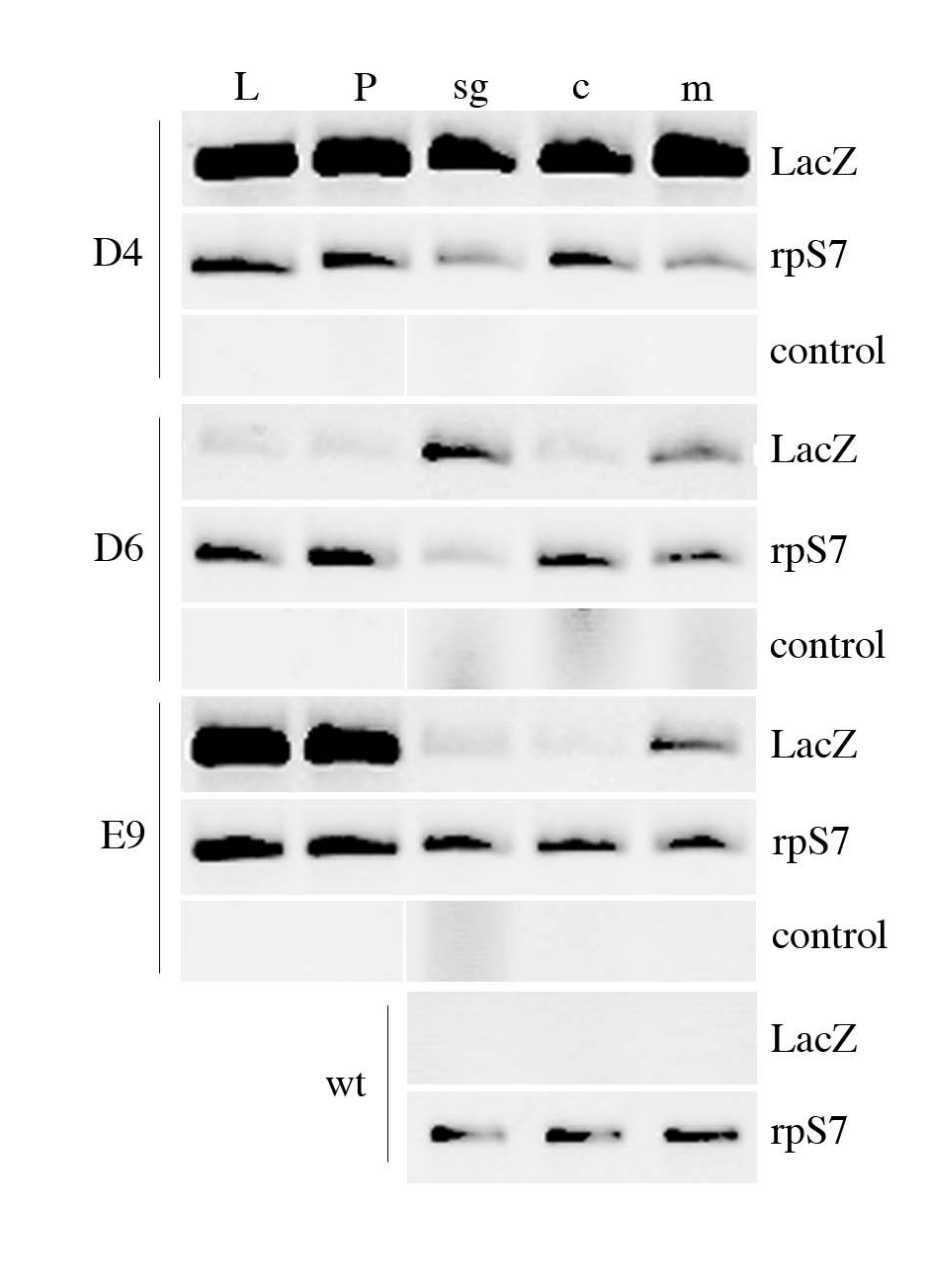
**Developmental- and tissue-specific *LacZ *expression analysis of three *An. gambiae *transgenic lines**. Total RNA from wild-type and transgenic mosquitoes was used to synthesize cDNA which was then amplified by PCR; as a control, PCR amplification of RNA templates without Reverse Transcriptase treatment was performed. The transgenic lines/families analyzed are indicated on the left. LacZ, RT-PCR amplification with *LacZ-bghT *specific primers, 35 cycles; rpS7, RT-PCR amplification with *rpS7*-specific primers, 25 cycles in order to keep the amplification below the saturation level; control, PCR amplification with *LacZ-bghT*-specific primers, 35 cycles. L, third and fourth instar larvae; P, pupae; sg, adult female salivary glands; c, carcasses (whole female body without salivary glands); m, males.

Stage and tissue expression analysis indicated that each of the three transgenic families analyzed exhibited a different temporal and spatial expression pattern of reporter gene expression (Fig. 2). Only in group D6 (carrying three copies of the transgene), were *LacZ *transcripts detected specifically in the adult female salivary glands and not in the female carcasses. However, reporter gene expression was also observed in males, indicating that sex-specificity of expression was not conserved. In group D4, which carries multiple copies of the transgene, *LacZ *transcripts were detected in all developmental stages. As such, it is likely that at least one transgene copy comes under the effect of surrounding genomic region, which confers a constitutive pattern of expression. In E9 line, carrying a single insertion, the *LacZ *gene was found highly expressed during larval and pupal stages and also clearly detectable in adult males, whereas expression in female salivary glands was barely detectable. This expression pattern is also probably conferred by 'position effect' of transgene insertion.

In conclusion, we report the efficient genetic transformation of *An. gambiae *and the characterization of an extended regulatory region of the salivary gland-specific *AgApy *gene. The transformation frequency (i.e. the percentage of G_0 _survivors producing fluorescent offspring) was estimated between 4 and 18% taking into consideration the potential occurrence of integration events early or late during germ-line development and the possible segregation of multiple insertions on different chromosomes. Since 71 G_0 _adults were batch mated, a minimum of 3 founders (two from group D and one from group E) identified and 13 distinct genotypes differentiated by Southern analysis, we calculated that from 3 to 13 independent integration events might have occurred. This transformation frequency is significantly higher as compared to those previously reported in primary research articles respectively by Kim W. and colleagues (1.2%, with 2 independent insertions out of 163 G_0 _adults) and Grossman G.L. and colleagues (0.6%, with only one transgenic founder out of 172 G_0 _crossed) [8,9]. Indeed, our report represents the first research paper validating the improvements and modifications introduced in the last few years and reviewed by Lobo N.F. and colleagues, where a transformation frequency range between 5 and 17% is observed [19]. We should also note the high number of multiple integrations obtained in our experiment (eight out of thirteen transgenic pedigrees). Insertion of multiple copies of the transgene is not always desirable because it can complicate line analysis and interpretation of the results, particularly since advanced genetic tools such as balancer chromosomes are not available for mosquitoes. It is widely documented that arthropod transformation by *piggyBac *yields multiple genomic insertions of the transgene [20]. The variability of its occurrence in different transformation experiments may depend from several factors and, among these, a primary role may be played by the ratio between transposon and helper plasmid and the timing (and temperature) of injections in relation to embryo development. However, there appears to be no simple correlation between transposon/helper ratio and occurrence of multiple insertions in the transformation experiments of anopheline mosquitoes documented to date (see Additional file 4).

Several hypotheses can be made to explain the lack of correspondence between the endogenous expression profile of the *AgApy *gene and the weak expression achieved in our transgenic mosquitoes (e.g. the construct misses enhancers or other regulatory regions, transcript instability or poor translational level), however it would remain speculative. Certainly, the results obtained both in *An. stephensi *[12] and in *An. gambiae *indicate that the control of sex- and tissue-specific expression by the *AgApy *promoter is more complex than originally anticipated. The selection of a longer portion of the *AgApy *promoter in comparison to the one analyzed in *An. stephensi *mosquitoes and the genetic transformation of the endogenous organism did not improve the efficacy of the putative *AgApy *promoter in transgenic insects. In particular, while a basal stage- and tissue-specific transcriptional activity is observed at least in one *An. gambiae *transgenic family, elements able to confer the typical strong and female-specific expression profile are again lacking. In conclusion, the *AgApy *salivary gland promoter described here, which is the only one examined in *An. gambiae *so far, was not capable to drive the expected strong tissue-specificity of expression, although it may be still useful when low levels of expression of the transgene are needed. Further efforts have to be addressed toward the identification and characterization of a strong salivary glands specific promoter in transgenic *An. gambiae*. The use of classical transposon-mediated approach in combination with insulators [21] or with site-specific integrases [22] to minimize variation produced by position effect would enhance research focused on this topic.

## Competing interests

The authors declare that they have no competing interests.

## Authors' contributions

FL, GJL, BA, AL and MC participated in the design of the study and of the experiments. FL, GJL and AL performed the experiments. FL, GJL and BA analyzed the data. FL, GJL and BA wrote the manuscript. All authors read and approved the final manuscript.

## Supplementary Material

Additional file 1 **Primers list**. This file contains the nucleotide sequences of the primers used in the work. Click here for file

Additional file 2 ***An. gambiae *****embryos injection protocol**. In this file is reported the detailed protocol adopted in this work to microinject *An. gambiae *embryos. Click here for file

Additional file 3 ***LacZ *****expression analysis in transgenic adult mosquitoes**. In this file is documented the comparison by RT-PCR between *LacZ *expression in transgenic *An. gambiae *and in transgenic *An. stephensi *mosquitoes, transformed with a shorter fragment of the same *AgApy *promoter. Click here for file

Additional file 4 ***piggyBac*****-mediated genetic transformation of Anophelinae mosquitoes**. A table listing the reports to date available in literature of genetic transformation of mosquitoes of the sub-family Anophelinae with *piggyBac*-based constructs is presented. Essential features from each transormation analysis are compared and references are indicated. Click here for file
