# Supplementary material for: Analysis of apyrase 5' upstream region validates improved Anopheles gambiae transformation technique
Source: BMC Res Notes. 2009 Feb 19;2:24. doi: 10.1186/1756-0500-2-24 (PMC2669092; doi:10.1186/1756-0500-2-24)
Supplement: Additional file 1 — Primers list. This file contains the nucleotide sequences of the primers used in the work. [file 1756-0500-2-24-S1.pdf]

Additional file 1

Primers list

Primers used for pBac(3xP3RED)AgApy plasmid construction:

ApyForKpn, 5’-CTAGGGTACCGAGTGCAAGGGAAGATGTGC-3’;

ApyRevRI, 5’-CTAGGAATTCCACGCTTCGCAGAGATTAC-3’.

Primers used to generate the pBac probes (pBacL and pBacR, spanning the pBac arms) by PCR:

pBacRF1, 5’-CAGGCCAGTGGGAACATC-3’;

pBacRR, 5’-GTACGTTAAAGATAATCATGCG-3’;

pBacLF1, 5’-CCGTCGCTGTGCATTTAGGA-3’;

pBacLR, 5’-TGACAATGTTCAGTGCAGAGAC-3’.

Primers used for RT-PCR analyses:

LacZ/bghT:

LBF, 5’-AGGCACATGGCTGAATATCG-3’;

LBR, 5’-GATGGCTGGCAACTAGAAGG-3’;

rpS7:

S7F, 5’-GGCGATCATCATCTACGTGC-3’;

S7R, 5’-GTAGCTGCTGCAAACTTCGG-3’;

LacZ:

LacZ-F1, 5’-TCAATCCGCCGTTTGTTCC-3’;

LacZ-R2, 5’-GTTCAGACGTAGTGTGACGC-3’;

DsRed:

RedF, 5’-TCCAAGGTGTACGTGAAGCA-3’;

RedR, 5’-CCCATGGTCTTCTTCTGCAT-3’.
