# Supplementary material for: Analysis of apyrase 5' upstream region validates improved Anopheles gambiae transformation technique
Source: BMC Res Notes. 2009 Feb 19;2:24. doi: 10.1186/1756-0500-2-24 (PMC2669092; doi:10.1186/1756-0500-2-24)
Supplement: Additional file 2 — An. gambiae embryos injection protocol. In this file is reported the detailed protocol adopted in this work to microinject An. gambiae embryos. [file 1756-0500-2-24-S2.pdf]

Additional file 2

An. gambiae *embryos injection protocol*

To prepare embryos, 10-15 female mosquitoes (G3 strain), 48-96 hours post-blood-feeding, were allowed to oviposit for a 20 minute period under insectary conditions. The collected embryos were left to mature for approximately another 30 minutes, then moved to room temperature and finally aligned in parallel against the flooded edge of moistened (25mM NaCl) filter paper on a glass microscope slide. Following alignment and withdrawal of the filter paper, embryos were briefly (20-30 seconds) air dried and transferred to a coverslip via double-sided sticky tape (3M #415). The embryos were rapidly covered in 25mM NaCl to prevent further desiccation and then injected under 200X magnification using inverted microscopy and quartz glass micropipettes created with a Sutter P2000 puller. Following the injection set, the eggs were left on the coverslip and submerged in a large volume of de-ionized water (200 – 300ml). Hatchlings were collected over the following 2-4 days; pupae were sexed and G_0 adults crossed to wt (wild type) at a ratio of 1:4 for G_0 males:wt females and of 1:5 for G_0 females:wt males. The resultant first and second instar larval progeny were initially screened *en masse* in small batches using a Nikon Stereoscope (SMZ1000) fitted with epi-fluorescence and a DsRed filter set (Nikon).
