# Supplementary material for: Analysis of apyrase 5' upstream region validates improved Anopheles gambiae transformation technique
Source: BMC Res Notes. 2009 Feb 19;2:24. doi: 10.1186/1756-0500-2-24 (PMC2669092; doi:10.1186/1756-0500-2-24)
Supplement: Additional file 3 — LacZ expression analysis in transgenic adult mosquitoes. In this file is documented the comparison by RT-PCR between LacZ expression in transgenic An. gambiae and in transgenic An. stephensi mosquitoes, transformed with a shorter fragment of the same AgApy promoter. [file 1756-0500-2-24-S3.pdf]

Additional file 3

LacZ expression analysis in transgenic adult mosquitoes

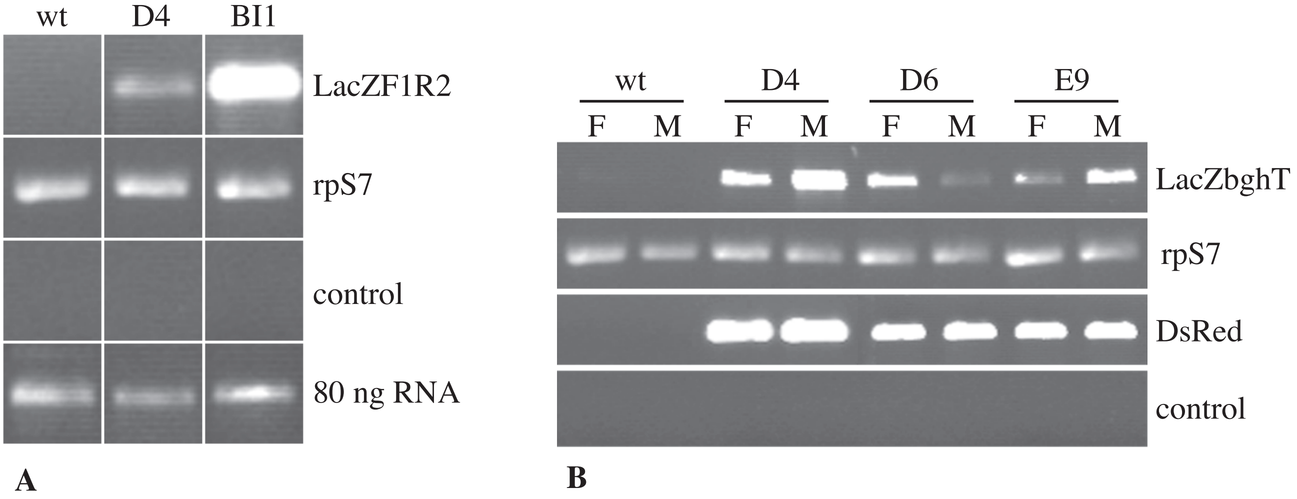

Panel A: *LacZ* expression analysis in transgenic *An. gambiae* (line D4) and in transgenic *An. stephensi* line BI1, carrying a shorter fragment of the *AgApy* promoter [12].
RNA from total females was extracted, quantified, DNAseI treated, tested by PCR for genomic contamination (control, PCR without RT using rpS7 primers pair, 35 cycles) and then used for RT-PCR amplification with the primers pairs indicated on the right. RpS7 normalization (rpS7) was performed at 25 cycles so that amplification products were not saturated; LacZ (LacZF1R2) amplification was performed at 35 cycles using the primers pair LacZF1R2, previously used for the analysis of transgenic *An. stephensi* [12]. The same amount of DNAseI treated RNA (80 ng) used in each amplification reaction was separated by agarose gel electrophoresis and stained with ethidium bromide as further loading control.

To improve the amplification efficacy of *LacZ* for further experiments in transgenic *An. gambiae*, a new primers pair was designed in order to perform the tissue and stage expression analysis detailed in Fig. 2.

Panel B: *LacZ* expression analysis in transgenic *An. gambiae* females and males.
The new primers pair (LacZbghT) was tested by RT-PCR on total female (F) and male (M) RNA samples extracted from wild-type (G3 strain, wt) and transgenic (lines D4, D6 and E9) mosquitoes. RNA was prepared as described above; control PCR (control) and normalization reactions (rpS7) were performed as described above; LacZ (LacZbghT) amplification was performed at 35 cycles using the primers pair LacZbghT; DsRed amplification (DsRed) was performed at 35 cycles.

Using the LacZbghT primers pair we have been able to obtain more efficient amplification of LacZ transcript from transgenic *An. gambiae*. This primers pair was therefore used for the tissue and stage analysis of the transgenic lines described in Fig. 2. The relative intensity of the LacZ amplification products among the *An. gambiae* transgenic lines is consistent with the *LacZ* expression profile shown in Fig. 2. Moreover, the intensity of the DsRed amplifications is compatible with the different marker gene copies carried by each transgenic line.
