# Supplementary material for: Analysis of apyrase 5' upstream region validates improved Anopheles gambiae transformation technique
Source: BMC Res Notes. 2009 Feb 19;2:24. doi: 10.1186/1756-0500-2-24 (PMC2669092; doi:10.1186/1756-0500-2-24)
Supplement: Additional file 4 — piggyBac-mediated genetic transformation of Anophelinae mosquitoes. A table listing the reports to date available in literature of genetic transformation of mosquitoes of the sub-family Anophelinae with piggyBac-based constructs is presented. Essential features from each transormation analysis are compared and references are indicated. [file 1756-0500-2-24-S4.pdf]

Additional file 4

piggyBac-*mediated genetic transformation of Anophelinae mosquitoes*

**Author**[1]	**Year**[2]	**Species**[3]	**N° lines**[4]	**N° lines, N° insertions**[5]	**pBac:Helper**[6]
Lombardo F. [this paper]		*gambiae*	13	5, I<br>4, II<br>3, III<br>1, ≥IV	3.5:1.5
Li C.	2008	*stephensi*	3	2, I<br>1, II	2.5:1.5
Chen X.G.	2007	*stephensi*	2	2, I	ref. Ito J. 2002
Chen X.G.	2007	*stephensi*	2	1, III<br>1, ≥IX	ref. Ito J. 2002
Nirmala X.	2006	*stephensi*	2	1, IV<br>1, ≥V	ref. Ito J. 2002
Catteruccia F.	2005	*stephensi*	5	single and multiple	4:1
Abraham E.G.	2005	*stephensi*	4	4, I	2.5:1.5
Kim W. [9]	2004	*gambiae*	2	1, I<br>1, II	2:1
Perera O.P.	2002	*albimanus*	10	3, I<br>1, II<br>1, III<br>4, IV<br>1, VI	1.5:1
Ito J.	2002	*stephensi*	4	4, I	2.5:1.5
Nolan T.	2002	*stephensi*	7	5, I<br>1, II<br>1, V	4:1
Moreira L.A.	2002	*stephensi*	4	4, I	2.5:1.5
Grossman G.L. [8]	2001	*gambiae*	1	1, I	2.5:1

A list of the reports to date available about genetic transformation of mosquitoes belonging to the sub-family Anophelinae with *piggyBac*-based constructs is presented in the Table, and few specific features from each work are compared.

1. The first author of the report.
2. The year of publication of the report.
3. The Anophelinae species transformed.
4. The number of transgenic lines reported from the authors.
5. The number of insertions (in roman numbers) carried by one or more transgenic line (arabic numbers).
6. The ratio between the amount of *piggyBac*-based construct and transposase-encoding helper plasmid used, as described in the Materials and Methods sections, or the cited reference.

References cited in the Table listed in alphabetic order (square brackets refer to publications in the manuscript bibliography).

Abraham EG, Donnelly-Doman M, Fujioka H, Ghosh A, Moreira L, Jacobs-Lorena M: **Driving midgut-specific expression and secretion of a foreign protein in transgenic mosquitoes with AgAper1 regulatory elements.** *Insect Mol Biol* 2005, **14**(3):271-279.

Catteruccia F, Benton JP, Crisanti A: **An Anopheles transgenic sexing strain for vector control.** *Nat Biotechnol* 2005, **23**(11):1414-1417.

Chen XG, Marinotti O, Whitman L, Jasinskiene N, James AA: **The Anopheles gambiae vitellogenin gene (VGT2) promoter directs persistent accumulation of a reporter gene product in transgenic Anopheles stephensi following multiple bloodmeals.** *Am J Trop Med Hyg* 2007, **76**(6):1118-1124.

Chen XG, Zhang YJ, Zheng XL, Wang CM: **Robust and regulatory expression of defensin A gene driven by vitellogenin promoter in transgenic Anopheles stephensi.** *Chinese Science Bulletin* 2007, **52**(14):1964-1969.

Ito J, Ghosh A, Moreira LA, Wimmer EA, Jacobs-Lorena M: **Transgenic anopheline mosquitoes impaired in transmission of a malaria parasite.** *Nature* 2002, **417**(6887):452-455.

Li C, Marrelli MT, Yan G, Jacobs-Lorena M: **Fitness of transgenic Anopheles stephensi mosquitoes expressing the SM1 peptide under the control of a vitellogenin promoter.** *J Hered* 2008, **99**(3):275-282.

Moreira LA, Ito J, Ghosh A, Devenport M, Zieler H, Abraham EG, Crisanti A, Nolan T, Catteruccia F, Jacobs-Lorena M: **Bee venom phospholipase inhibits malaria parasite development in transgenic mosquitoes.** *J Biol Chem* 2002, **277**(43):40839-40843.

Nirmala X, Marinotti O, Sandoval JM, Phin S, Gakhar S, Jasinskiene N, James AA: **Functional characterization of the promoter of the vitellogenin gene, AsVg1, of the malaria vector, Anopheles stephensi.** *Insect Biochem Mol Biol* 2006, **36**(9):694-700.

Nolan T, Bower TM, Brown AE, Crisanti A, Catteruccia F: **piggyBac-mediated germline transformation of the malaria mosquito Anopheles stephensi using the red fluorescent protein dsRED as a selectable marker.** *J Biol Chem* 2002, **277**(11):8759-8762.

Perera OP, Harrell IR, Handler AM: **Germ-line transformation of the South American malaria vector, Anopheles albimanus, with a piggyBac/EGFP transposon vector is routine and highly efficient.** *Insect Mol Biol* 2002, **11**(4):291-297.
